# When Diet Trends Go Viral: Cutaneous Manifestations of Social Media-Driven Fad Diets and Supplements

**DOI:** 10.7759/cureus.86334

**Published:** 2025-06-19

**Authors:** Andres D Parga, Hannah Coven

**Affiliations:** 1 Medicine, HCA Florida Oak Hill Hospital, Brooksville, USA; 2 Nutrition and Dietetics, Arizona State University, Tempe, USA

**Keywords:** dermatology, dietary supplements, fad diets, nutrition, nutritional deficiency, public health, skin disorders

## Abstract

The rise of fad diets and unregulated supplement use, amplified by social media and aggressive marketing, has dramatically shifted public attitudes toward nutrition and health. This new landscape is associated with a growing spectrum of dermatologic presentations, as the skin frequently serves as an early indicator of both nutritional deficiencies and toxicities. Popular dietary trends, such as ketogenic, carnivore, and raw vegan regimens, have been linked to cutaneous disorders, including prurigo pigmentosa, scurvy-like eruptions, hair loss, xerosis, and pigmentary changes. In parallel, the overuse of supplements like niacin, selenium, and zinc is increasingly implicated in cases of dermatologic toxicity, presenting as flushing, dermatitis, alopecia, and nail dystrophy. Fitness-oriented supplements, particularly whey protein, are now recognized contributors to acne flares, likely via insulin and IGF-1 pathways. Given the high prevalence of health misinformation online and the lack of regulation surrounding dietary supplements, dermatologists and allied clinicians must prioritize thorough dietary and supplement histories, recognize the skin’s role as an early warning system for nutritional imbalance, and provide evidence-based patient counseling. A multidisciplinary approach that integrates clinical vigilance, interdisciplinary education, and public health advocacy will be crucial for reducing the burden of nutrition-related skin disease and promoting safe, effective dietary practices. The clinical recognition of these diet- and supplement-induced dermatoses is complicated by their overlap with more common inflammatory or autoimmune skin diseases, often leading to misdiagnosis and delayed management of the underlying nutritional etiology.

## Introduction and background

The intersection of diet, supplements, and dermatologic health is increasingly important in contemporary clinical practice, particularly as digital platforms influence public perceptions of nutrition and wellness [1,2]. The rise of online communities, influencer-driven content, and direct-to-consumer marketing has transformed how individuals access dietary information and select nutritional regimens or supplements [3]. While these channels can improve health literacy and support healthy choices, they can also facilitate the rapid spread of misinformation, particularly regarding restrictive diets and supplement use [1,4]. Recent analyses indicate that a significant proportion of nutrition-related content on platforms such as YouTube, Instagram, and TikTok may lack scientific rigor or present unverified claims [1,4]. Recent evidence highlights the significant influence of social media on shaping dietary behaviors, particularly among adolescents and young adults. Platforms such as TikTok, with over one billion monthly users primarily aged 10 to 29, significantly amplify nutrition misinformation due to algorithm-driven content prioritizing engagement over accuracy. A recent study evaluating nutrition-related TikTok posts found that 55% lacked evidence-based information, and 90% failed to adequately discuss the risks and benefits of the nutritional advice provided. Moreover, only 36% of analyzed posts were classified as completely accurate, highlighting the extent of misinformation prevalent on popular social media channels [5]. This variability in information quality, combined with commercial interests, has contributed to the normalization, and, in some instances, glamorization of extreme dietary patterns and unregulated supplement use [6,7]. Against this backdrop, the skin serves as a sensitive and visible marker of underlying nutritional status, often manifesting changes before systemic symptoms become apparent [8-10]. A range of cutaneous findings, including acute rashes, pigmentary alterations, telogen effluvium, nail dystrophies, and chronic dermatitis, have been described in association with restrictive dietary trends (e.g., ketogenic, vegan, carnivore, and paleo diets) and unsupervised supplement use [11,12]. These presentations may reflect both classic deficiency syndromes (such as scurvy, pellagra, or zinc deficiency) and more recently recognized entities, such as prurigo pigmentosa from ketogenic diets or selenosis-induced alopecia related to supplement misuse [13-15]. Importantly, the ease of access to online health information is a double-edged sword: while it can empower patients, it may also increase exposure to low-quality or misleading content, an issue that has been recognized as a broader public health concern [4,16]. In dermatology, the visual impact of skin disease and the popularity of "before-and-after" imagery further amplify the role of digital trends in shaping patient behaviors and expectations. Although causality between social media exposure and disease outbreaks is complex and multifactorial, there are documented cases where misinformation or commercial promotion has contributed to dietary or supplement-related adverse events [7,17,18]. Given these challenges, it is essential for clinicians, particularly dermatologists, to recognize the spectrum of cutaneous manifestations linked to contemporary dietary practices, to provide balanced, evidence-based counseling, and to support improved digital health literacy among patients [1,16]. This multidisciplinary, patient-centered approach will be increasingly important in bridging the gap between evolving digital culture and safe, science-based nutrition and dermatologic care.

## Review

Materials and methods

This narrative review synthesizes current evidence on the dermatologic consequences of fad diets and unregulated supplement use, with a particular focus on the role of social media and digital health information in shaping dietary behaviors. To identify relevant literature, a comprehensive search of electronic databases, including PubMed, MEDLINE, Embase, and Google Scholar, was conducted for articles published between January 2010 and May 2025. Search terms included combinations of “fad diet,” “ketogenic,” “carnivore,” “vegan,” “plant-based diet,” “supplement,” “selenium,” “niacin,” “zinc,” “whey protein,” “acne,” “hair loss,” “dermatologic toxicity,” “skin disease,” “social media,” “TikTok,” “Instagram,” “nutrition misinformation,” and “digital health literacy.” Additional references were identified through the bibliographies of key articles and recent systematic reviews. Eligible studies included peer-reviewed original research, systematic reviews, meta-analyses, case reports, and narrative reviews addressing the relationship between specific dietary patterns, supplement use, and cutaneous manifestations. Both adult and adolescent populations were considered, with special attention given to recent literature reflecting the influence of contemporary social media trends. Non-human studies, articles lacking dermatologic endpoints, and sources without full-text access were excluded. Key data were extracted from selected studies, including the type of diet or supplement, study design, population demographics, dermatologic outcomes, and underlying nutritional or toxicologic mechanisms. Particular emphasis was placed on recent surveys and qualitative analyses evaluating the prevalence, content quality, and behavioral impact of nutrition and diet-related information on social media platforms such as TikTok and Instagram. Studies quantifying user engagement, the role of influencers, and the accuracy of digital nutrition advice were prioritized when available. Although much of the available evidence consisted of observational studies, case series, and self-reported survey data, findings from randomized controlled trials and systematic reviews were highlighted when relevant. Methodological limitations and the risk of bias were acknowledged, especially regarding studies utilizing social media analytics and self-reported behavior. The selection and synthesis of evidence were independently reviewed by all authors to ensure comprehensive and balanced coverage of key themes, with discrepancies resolved by consensus. This review adheres to current recommendations for transparency in narrative reviews, including clear justification for included literature and a discussion of evidence limitations.

Fad diets and dermatologic effects

Fad diets have long been a part of the popular nutrition landscape, with their dermatologic manifestations well-described across multiple eras and media, from word-of-mouth and print to television and, more recently, digital platforms (Table 1). While the underlying risks of nutrient deficiency, excess, or imbalance are longstanding, shifts in the modes of information sharing, particularly through social media and online communities, have increased the visibility and rapid adoption of novel dietary trends. For example, a 2022 qualitative analysis of 1,000 top-viewed TikTok videos tagged with nutrition or diet-related hashtags (each exceeding one billion views) found that nearly 44% of posts focused on weight loss and only 1.4% offered advice from registered dietitians. Less than 3% of these highly-engaged posts promoted weight-inclusive messaging, while the vast majority glorified rapid weight loss and extreme dietary practices [19]. Surveys of college-aged women reveal that over 98% actively use Instagram, and nearly half spend at least three to four hours per day on social media, encountering food and nutrition content multiple times daily. Approximately 69% report that social media directly influences their food choices, with more than half having eliminated foods from their diet based on content encountered online [20]. Furthermore, the “#weightloss” hashtag alone accumulated nearly 10 billion views at the time of analysis, highlighting the massive reach and potential influence of these messages among adolescents and young adults, who are the platform’s primary users. Notably, 27% of surveyed respondents reported participating in fad diets, such as ketogenic or carnivore regimens, after seeing related posts online, while 80% encountered supplement promotions, and 93% saw weight-loss strategy content on their social feeds [20]. However, current data do not support a clear epidemiologic increase in diet-related skin disease attributable solely to digital media. Rather, the available literature is largely comprised of surveys, case reports, small series, and expert opinion, underscoring both the enduring nature of these conditions and the need for ongoing clinical vigilance. This section summarizes the key dermatologic risks associated with major dietary patterns, drawing on established literature and highlighting the importance of a detailed dietary history in dermatologic practice. It is important to note that most claims surrounding fad dietary supplements lack robust evidence from randomized double-blinded controlled clinical trials, underscoring the need for caution in clinical practice.

**Table 1:** Major Fad Diets and Associated Dermatologic Risks

Diet Type	Key Skin Findings	Main Nutrient Risks	Typical Onset	Notes
Ketogenic	Prurigo pigmentosa, xerosis, rash, hair loss	Vitamin C, magnesium, selenium, zinc	1–4 weeks	“Keto rash” can be misdiagnosed
Vegan/Raw Vegan	Alopecia, cheilitis, glossitis, dermatitis, hyperpigmentation	B12, iron, biotin, essential fatty acids, zinc	Weeks–months	Poorly planned diets increase risk
Carnivore/Zero-Carb	Scurvy-like rash, delayed wound healing, brittle hair	Vitamin C, fiber, magnesium, calcium, folate	Months	Scurvy risk is real, especially w/o organ meats
Juice Cleanse	Xerosis, brittle nails, alopecia	Protein, biotin, essential fatty acids	Weeks–months	Rapid weight loss triggers telogen effluvium
Paleo	Dermatitis, xerosis, and possible calcium deficiency	Calcium, vitamin D	Variable	Excludes dairy/grains
Mediterranean	Lower risk of inflammatory dermatoses	Generally low risk	—	Protective against acne, psoriasis
Gluten-Free (non-celiac)	Xerosis, possible nutrient deficiencies	Iron, B vitamins	Variable	Unnecessary restrictions can backfire

Ketogenic and very-low-carbohydrate diets

The ketogenic diet, characterized by severe carbohydrate restriction and high fat intake, has soared in popularity, largely propelled by online communities and influencer-driven content promising rapid weight loss, improved metabolic health, and neurological benefits [6]. However, this dietary pattern has distinct, often underrecognized, dermatologic risks (Table 2).

**Table 2:** Prurigo Pigmentosa in Ketogenic Diets PIH: post-inflammatory hyperpigmentation.

Clinical Feature	Findings
Onset	6 days–4 months (mean 2–4 weeks after starting diet)
Distribution	Trunk, neck, and sometimes face
Histology	Early: neutrophilic infiltrate; late: melanophages, parakeratosis
Management	Carbohydrate reintroduction, tetracyclines if persistent
Prognosis	Excellent, but PIH common

Prurigo pigmentosa ("keto rash") is a hallmark complication, presenting as intensely pruritic, erythematous papules and plaques in a reticulated pattern over the trunk, neck, and sometimes face [13,21]. Onset typically occurs within one to four weeks of entering ketosis, and resolution is rapid with carbohydrate reintroduction or discontinuation of the diet. Histopathology reveals a progression from superficial perivascular neutrophilic infiltrates (early) to melanophages, parakeratosis, and spongiosis (later stages) [13,21]. Ketone bodies are believed to promote neutrophil-mediated perivascular inflammation and skin autophagy. Other triggers include diabetes, fasting, pregnancy, and bariatric surgery [13,21]. Clinical awareness of keto rash remains low, leading to frequent misdiagnosis as eczema, contact dermatitis, or other rashes. A routine diet history is essential for any new-onset reticulated, pruritic rash.

Some evidence supports the benefit in psoriasis and systemic inflammation with properly supervised ketogenic or hypocaloric diets [22,23]. Mechanisms include suppression of the nucleotide-binding oligomerization domain, leucine-rich repeat, and pyrin domain containing (NLRP)3 inflammasome, reduced pro-inflammatory cytokines (TNF-α, IL-6), and improved lipid profiles. However, these effects are not universal and may be offset by the risk of nutritional deficiencies, acidosis, or kidney stones with prolonged unsupervised use [6,11]. Additionally, micronutrient deficiency syndromes have been observed in some cases. Metabolic acidosis, nephrolithiasis, and classic vitamin/mineral deficiencies (zinc, selenium, magnesium, vitamin C) have been reported, especially with unsupervised or extended adherence [6,10].

Vegan and plant-based diets

While well-planned vegan diets are generally safe, extreme plant-based or poorly supplemented diets carry risk for specific nutrient deficiencies and their dermatologic sequelae (Table 3) [24,25]. Classic deficiency findings include angular cheilitis (B2, iron), glossitis (B12, iron, folate), erythematous or seborrheic-like dermatitis (B2, B6), phrynoderma (vitamin A, essential fatty acids), and diffuse alopecia (iron, protein, zinc, biotin) [10,25]. Protein and essential fatty acid deficiency can lead to xerosis, impaired barrier function, brittle nails, and increased risk of eczematous rashes. Vitamin B12 and iron deficiency are among the most common, resulting in hyperpigmentation, glossitis, and telogen effluvium [10]. Another interesting aspect of vegan diets is the occasional case of beta-carotenemia. Overconsumption of carotene-rich fruits/vegetables may cause yellow-orange skin discoloration, especially in raw vegan diets [26].

**Table 3:** Key Dermatologic Manifestations in Vegan Diets EFA: essential fatty acids.

Nutrient Deficiency	Skin Finding
Vitamin B12, Iron	Hyperpigmentation, glossitis, alopecia
Vitamin B2, B6	Cheilitis, seborrheic dermatitis
Protein/EFA	Xerosis, impaired healing
Biotin	Periorificial dermatitis, hair loss

Appropriately balanced vegan and plant-based diets may reduce the risk or severity of acne, hidradenitis suppurativa, psoriasis, and atopic dermatitis, attributed to reduced intake of dairy, animal fats, and high-glycemic foods, and an increase in fiber, antioxidants, and anti-inflammatory nutrients [22,24]. Moreover, vegan diets typically result in lower circulating IGF-1 levels, which, while potentially protective against certain malignancies, could also impair dermal repair and collagen synthesis, particularly in nutritionally compromised individuals.

Raw veganism and juice cleanses

Promoted for their "detoxifying" effects and skin benefits, these diets can be high in antioxidants but dangerously low in protein, essential fatty acids, and certain vitamins/minerals [8,9]. Protein and biotin deficiency (diffuse alopecia, brittle nails), essential fatty acid deficiency (xerosis, impaired barrier, increased irritant/allergic dermatitis), and beta-carotenemia (yellow-orange discoloration) are all well-documented, especially with long-term restrictive adherence [8,26]. Juice cleanses are particularly low in protein/fat, impairing healing and barrier synthesis, increasing the risk of chronic xerotic eczema [9,16].

Carnivore and zero-carbohydrate diets

These extreme diets, eliminating virtually all plant-derived foods, are heavily promoted online across social media platforms like TikTok with claims of anti-inflammatory and autoimmune benefits [27,28]. However, the clinical evidence base is sparse, and significant risks exist. Vitamin C deficiency and scurvy: With minimal ascorbic acid intake, even marginal deficiency may produce perifollicular hemorrhage, follicular hyperkeratosis, delayed wound healing, and, in severe cases, frank scurvy [14,25]. Several cases have been documented in adults following strict carnivore regimens, rapidly resolving with vitamin C supplementation. Gut microbiome disruption: Lack of dietary fiber and plant polyphenols reduces gut microbial diversity and short-chain fatty acid production, impairing skin barrier and immunity, and increasing risk of atopic dermatitis and other inflammatory dermatoses [11,29]. Other deficiency syndromes: These include magnesium, potassium, thiamin, biotin, and essential fatty acid deficiencies, with corresponding risks for dry, scaly skin, hair thinning, onychoschizia, and impaired barrier recovery [26,27]. Self-reported survey data suggest a low prevalence of overt deficiency dermatoses in short-term adherents, but lack of clinical/laboratory confirmation and likely survivor/reporting bias limit these findings [28].

Mediterranean, paleo, and other popular diets

Robust evidence supports anti-inflammatory, antioxidant-rich effects with lower risk and severity of psoriasis, hidradenitis suppurativa, acne, and possibly skin cancer [6,22]. Emphasizes whole grains, extra virgin olive oil, nuts, fish, and vegetables. Paleolithic diet, which excludes grains, legumes, dairy-may reduce triggers of acne/eczema but carries risk for calcium and micronutrient deficiencies [6]. Furthermore, gluten-free diets, essential for those living with dermatitis herpetiformis and celiac disease, show emerging evidence for benefit in selected autoimmune dermatoses with proven gluten sensitivity [11,22].

Supplement overuse and skin toxicity

The widespread use and overconsumption of dietary supplements, often driven by social media and influencer marketing, have introduced unique risks for dermatologic toxicity. Many supplements, including selenium, niacin, zinc, and various protein powders, are associated with cutaneous side effects such as hair loss, rashes, nail changes, and pigmentary alterations. These manifestations may mimic or overlap with other skin conditions, making recognition and diagnosis challenging for clinicians. The most frequently implicated supplements and their characteristic dermatologic risks are summarized below (Table 4).

**Table 4:** Supplements Most Commonly Linked to Dermatologic Toxicity

Supplement/Ingredient	Dermatologic Risk	Typical Toxic Dose	Clinical Clues	Regulatory Notes
Selenium	Rapid alopecia, nail dystrophy, xerosis	>400–500 mcg/day	Painless, sudden hair loss; nail changes	High doses are often mislabeled
Niacin (B3)	Flushing, erythema, and exfoliative dermatitis	>500 mg/day	Red face/neck, itching	Common in “preworkout”
Zinc	Hypopigmentation, poor wound healing, brittle nails/hair	>40 mg/day	Pigment loss, nonhealing wounds	Induces copper deficiency
Copper (deficiency)	Fragile skin, depigmented hair, slow healing	Excess zinc suppresses	Pale skin, brittle hair, ulcers	Secondary to zinc overuse
Whey protein	Acne (face/trunk)	High-dose daily use	Onset/worsening after supplement	Dairy- or egg-based
Anabolic agents	Acne, alopecia, striae	Supra-physiologic doses	Severe cystic acne, balding	Often as unlisted ingredient

Selenium toxicity (selenosis)

Selenium, while essential for antioxidant defense and thyroid function, has a very narrow therapeutic window. Supplement overuse-intentionally or via mislabeling/adulteration-can result in selenosis, a syndrome with profound dermatologic and systemic consequences (Table 4). While social media trends can drive supplement overuse, the well-documented U.S. selenium toxicity outbreak was linked to misprescribing and labeling errors among alternative health practitioners, highlighting the multifactorial nature of supplement-related risks [17], presenting with rapid, painless scalp hair loss (sometimes progressing to complete baldness), dystrophic or discolored nails, and systemic features such as gastrointestinal (GI_ upset, fatigue, and characteristic “garlic breath.” Hair loss may begin within days to weeks of exposure, and nail changes often follow. Individual cases have arisen from excessive ingestion of “natural” selenium sources, notably paradise nuts (Lecythis ollaria). Consumption of 10-15 nuts per day for several weeks has led to dramatic alopecia and onycholysis, with laboratory selenium levels four to six times the upper limit of normal (Table 5) [18]. Selenosis may also manifest as xerosis, eczema, or desquamation; more severe intoxication causes neuropathy, metallic taste, and hepatotoxicity. Symptoms generally improve after cessation of exposure, but hair/nail regrowth is slow and may be incomplete [18].

**Table 5:** Common Features and Outbreaks of Selenosis GI: gastrointestinal.

Source	Clinical Clues	Lab Findings
Liquid supplement (misformulated)	Rapid alopecia, nail dystrophy, GI upset	Plasma Se > 400–500 mg/L
Paradise nuts (plant-based)	Diffuse alopecia, onycholysis, nail discoloration	Plasma Se > 500 mg/L
Beauty/biohacking supplements	Mixed symptoms, chronic or subacute	Elevated Se

Many beauty and “biohacking” supplements contain far more selenium than labeled; neither the Food and Drug Administration (FDA) nor most countries require premarket efficacy/safety review of supplements [7]. Overlapping supplement use and consumption of selenium-rich foods (e.g., Brazil nuts) can easily push intake above toxic thresholds. It is important in this digital era to suspect selenosis in unexplained, rapid-onset alopecia or nail changes-especially in supplement users. Measurement of plasma selenium is diagnostic; discontinuation is curative in most cases [17,18].

Niacin (vitamin B3) toxicity 

Niacin is a frequent component of fortified foods, energy drinks, and “preworkout” or bodybuilding supplements. While moderate supplementation is beneficial, excessive intake can cause distinctive acute and chronic dermatologic effects [8,30]. Acute niacin toxicity, also referred to as niacin flush, is a dose-dependent, prostaglandin D2-mediated erythema and warmth, affecting the face, neck, and upper chest. It is transient but uncomfortable and often reported after energy drinks or high-dose supplements [30]. In chronic high-dose use, more severe skin findings may appear, such as persistent erythema, exfoliative dermatitis, and generalized erythroderma. Additionally, high doses can lead to liver dysfunction, manifesting as jaundice, spider angiomata, or desquamation.

Overlap of multiple energy and performance supplements can result in unintentional niacin megadosing, especially in athletes and bodybuilders [31]. Dermatologists should ask about “hidden” niacin in fortified foods/drinks and preworkout supplements in patients with new-onset flush, erythema, or chronic dermatitis.

Zinc & copper imbalance

Zinc is widely marketed for immune support, acne, and hair loss-but excessive supplementation, particularly at high doses, can disrupt copper absorption and cause a secondary copper deficiency syndrome [9,32]. High-dose, chronic zinc intake (often self-prescribed) is increasingly common in athletes, “wellness” followers, and those using overlapping multivitamin blends. Dermatologic findings of copper deficiency syndrome include skin and hair hypopigmentation, depigmented or “washed-out” hair, skin fragility, poor wound healing, and persistent ulcers. Other systemic findings may include anemia, neutropenia, neuropathy, and ataxia (in advanced cases). On a biochemical level, zinc and copper share intestinal transporters; excessive zinc blocks copper uptake.

Unexplained pigmentary changes, nonhealing ulcers, or brittle hair/nails in anyone with supplement use should prompt zinc/copper measurement and a detailed history. Most cases resolve with copper replacement and stopping zinc.

Protein supplements and acne

Whey protein, ubiquitous in fitness, sports, and “wellness” culture, is a well-established dietary trigger for acne in predisposed individuals [33,34]. Whey protein increases insulin and IGF-1 signaling, which upregulate androgenic pathways, sebaceous gland activity, and keratinocyte proliferation-major contributors to acne pathogenesis [33]. Multiple case series and epidemiologic studies show a temporal link between whey protein initiation and onset/exacerbation of acne, especially on the trunk and face in adolescent/young adult males [34]. Resolution is typical after stopping whey supplementation.

Dermatologists need to inquire about protein supplement use in new or refractory acne-especially in young athletes or bodybuilders. Additionally, widespread use of collagen supplements, typically derived from marine or bovine sources, has gained popularity for improving skin elasticity and reducing aging signs. While preliminary evidence supports some benefits, excessive intake can lead to gastrointestinal disturbances and allergic reactions, underscoring the importance of moderation and medical supervision.

Performance enhancers and bodybuilding supplements

Beyond “basic” vitamins/minerals, many bodybuilding or energy supplements contain risky and poorly regulated ingredients, which may directly cause cutaneous toxicity or serve as markers of more serious systemic risk. In contrast, specific omega-3 fatty acid formulations, such as icosapent ethyl and omega-3-acid ethyl esters, are FDA-approved, primarily for hypertriglyceridemia, though emerging evidence suggests potential dermatologic benefits at therapeutic dosages ranging from 2-4 grams per day. Clinical evidence regarding omega-3 supplementation specifically for acne [35], however, remains preliminary and warrants further investigation. Supplements may contain or be adulterated with anabolic steroids, stimulants (DMAA), yohimbe, prohormones, and unlisted drugs [7,36]. Classic cutaneous adverse effects include: acne (increased sebum), androgenic alopecia, striae distensae, and increased infection risk. One study found that supplement users are significantly more likely to use other performance drugs, compounding risk [15]. Label inaccuracies and intentional adulteration are well documented. The Dietary Supplement Label Database (DSLD) and FDA warning lists are critical resources, but do not capture all risks [7].

In a patient with new-onset severe acne, striae, unexplained alopecia, or signs of virilization, it may be advisable to screen for bodybuilding supplement and anabolic agent use.

Hidden hazards: supplement misinformation and social media

Health misinformation is rampant. Up to half of all supplement-related advice on social media is inaccurate or misleading, especially regarding “detox” protocols, megadosing, and biohacking [4]. Misinformation drives real-world behavior, raising the risk for toxicity and dermatologic sequelae (Table 6). Dietary supplement overuse, hidden adulterants, and poor regulation create genuine risks for skin, hair, and nail disease. A careful dietary and supplement history is essential in any patient with new or unexplained cutaneous findings. Raising patient and provider awareness and advocating for stronger regulation and health literacy is critical in the era of online misinformation.

**Table 6:** Social Media Dietary Claims: Accuracy and Dermatologic Consequences GI: gastrointestinal.

Claim Type	Diet/Supplement	Reported Skin Outcome	Accuracy	Clinical Note
“Keto cures acne”	Ketogenic diet	Rash, prurigo pigmentosa	Misleading	May trigger rash, may help some acne
“Vegan clears your skin”	Vegan diet	Dermatitis, alopecia, pigment changes	Mixed	Benefit if well-planned, risk if not
“Carnivore stops eczema”	Carnivore diet	Scurvy-like rash, hair loss	False	Major risk of vitamin C deficiency
“Supplements for hair growth”	Biotin, zinc, selenium	Hair loss, nail dystrophy, rashes	Misleading	Overuse causes/worsens hair loss
“Whey protein builds muscle, no side effects”	Whey protein	Acne outbreaks, GI upset	Misleading	Acne exacerbation common

Public health & clinical implications

Clinicians must acknowledge that despite anecdotal associations, rigorous epidemiological data directly linking social media-driven dietary trends to an increased incidence of dermatologic diseases remain limited. Nonetheless, the clinical relevance of recognizing and managing these conditions remains essential. The explosion of restrictive diets and unregulated supplement use-driven largely by social media influencers and aggressive marketing-has produced a new set of public health challenges for dermatologists and allied clinicians. Systematic reviews demonstrate that a substantial proportion of diet and supplement-related content online is inaccurate or misleading, especially for fad diets, “detox” regimens, and unproven “miracle” supplements [4]. This misinformation is amplified within social media echo chambers, where influencer-driven trends normalize extreme dietary behaviors and supplement overuse, increasing the risk of both dermatologic toxicity and nutritional deficiency syndromes. Among the most concerning findings is that up to 87% of posts about drugs and supplements may include unsubstantiated or hazardous claims, with particular risks seen in content targeting young adults, athletes, and those pursuing rapid physical transformation. The lack of regulatory oversight for dietary supplements in many regions-including the United States, further exacerbates these risks. Products may contain excessive, mislabeled, or unlisted ingredients, and there is no requirement for premarket safety or efficacy review [7]. High-profile outbreaks, such as cases of acute selenosis linked to misformulated supplements or “natural” plant sources like paradise nuts, illustrate the serious dangers posed by poorly regulated products [17,18]. Clinicians must recognize that patients frequently consume multiple overlapping supplements and may be exposed to unintentional toxicities.

Dermatologists are uniquely positioned to recognize early cutaneous markers of nutritional pathology, ranging from prurigo pigmentosa related to ketogenic diets, selenosis-induced alopecia, to acne flares triggered by whey protein or other supplement use [9,21]. Classic presentations, such as rapid and painless scalp hair loss, nail dystrophy, or new-onset reticulated rashes, should prompt clinicians to obtain a comprehensive dietary and supplement history, including inquiry about social media influences and online trends. Early identification not only benefits individual patients by enabling prompt intervention (often with simple dietary or supplement cessation) but also has broader public health implications, allowing for recognition of emerging trends and potential outbreaks.

Effective management requires an interdisciplinary approach. Education for both dermatology and nutrition professionals should incorporate critical appraisal skills and digital health literacy, enabling clinicians to better counteract online misinformation and guide patients in evaluating dubious health claims [3,16]. Collaboration between dermatologists, dietitians, and primary care providers is essential for addressing complex cases, especially those involving restrictive eating patterns, supplement overuse, or suspected toxicity. Patient counseling should focus on the importance of balanced, whole-food-based dietary patterns, caution against unproven or excessive supplementation, and provide direction to reputable resources, such as the Dietary Supplement Label Database (DSLD), FDA Tainted Product List, and operation supplement safety (OPSS) High-Risk Supplement List. Patients should be warned that “natural” does not mean “safe,” and that even plant-based supplements can cause severe toxicity if misused or taken in excess.

**Figure 1:**
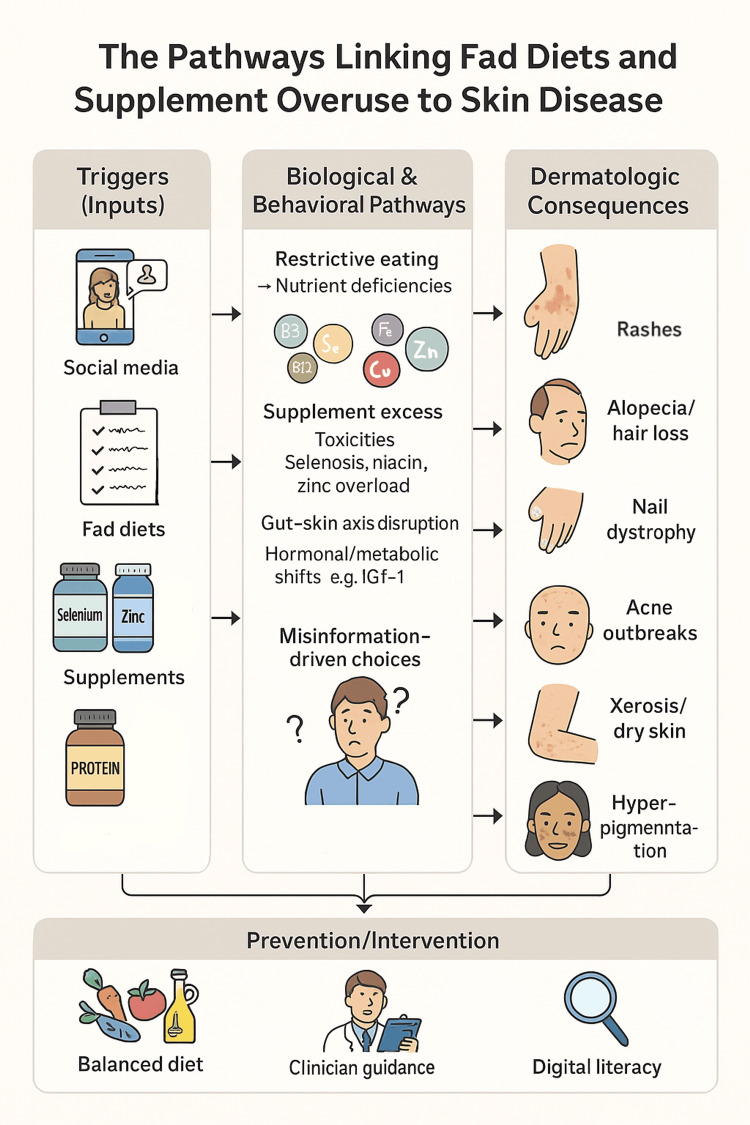
The Digital-to-Dermis Cascade: How Social Media Drives Diet-Related Skin Disease The multi-factorial pathways linking fad diets, supplement overuse, and misinformation to dermatologic disease. Social media and online trends drive restrictive or excessive nutrition behaviors, resulting in deficiencies or toxicities that manifest with a range of cutaneous findings. Prevention centers on balanced diet, patient education, and critical appraisal of online health information. Figure rendered by the authors using Procreate (2025).

Finally, clinicians should advocate for stronger regulatory oversight of the supplement industry and for public education campaigns that promote digital health literacy, particularly among adolescents and young adults who are the highest users of both fad diets and supplements (Figure 1) [37]. Advocacy efforts should include support for clear labeling, mandatory adverse event reporting, and community programs that address both nutrition misinformation and eating disorder risk. Clinicians should integrate structured nutritional screening into dermatologic assessments, collaborate closely with dietitians for complex cases, and employ targeted laboratory evaluations when dietary or supplement-related dermatologic disorders are suspected. Ultimately, the intersection of diet, supplements, and dermatology is a growing frontier for both clinical practice and public health, demanding vigilance, interdisciplinary collaboration, and a proactive approach to patient education and policy.

## Conclusions

Fad diets and unregulated supplement use, driven by social media and misinformation, are increasingly linked to dermatologic disease. The skin often signals early warning of nutritional deficiency or toxicity, making thorough dietary and supplement histories critical for diagnosis. Clinicians should educate patients on the risks of extreme diets and unproven supplements, emphasize balanced nutrition, and advocate for improved regulation and digital health literacy. Early recognition and multidisciplinary collaboration can help prevent serious cutaneous and systemic consequences of modern diet trends.
